# Acrylamide in potato crisps prepared from 20 UK-grown varieties: Effects of variety and tuber storage time

**DOI:** 10.1016/j.foodchem.2015.02.103

**Published:** 2015-09-01

**Authors:** J. Stephen Elmore, Adrian Briddon, Andrew T. Dodson, Nira Muttucumaru, Nigel G. Halford, Donald S. Mottram

**Affiliations:** a Department of Food and Nutritional Sciences, University of Reading, Whiteknights, Reading RG6 6AP, UK; b AHDB Potato Council, Sutton Bridge Crop Storage Research, East Bank, Sutton Bridge, Spalding, Lincolnshire PE12 9YD, UK; c Plant Biology and Crop Science Department, Rothamsted Research, Harpenden, Hertfordshire AL5 2JQ, UK

**Keywords:** Acrylamide, Reducing sugars, Free amino acids, Potato, *Solanum tuberosum*, Crisps, Colour, Maillard reaction, Storage, Cultivar

## Abstract

Twenty varieties of field-grown potato were stored for 2 months and 6 months at 8 °C. Mean acrylamide contents in crisps prepared from all varieties at both storage times ranged from 131 μg/kg in Verdi to 5360 μg/kg in Pentland Dell. In contrast to previous studies, the longer storage period did not affect acrylamide formation significantly for most varieties, the exceptions being Innovator, where acrylamide formation increased, and Saturna, where it decreased. Four of the five varieties designated as suitable for crisping produced crisps with acrylamide levels below the European Commission indicative value of 1000 μg/kg (Saturna, Lady Rosetta, Lady Claire, and Verdi); the exception was Hermes. Two varieties more often used for French fries, Markies and Fontane, also produced crisps with less than 1000 μg/kg acrylamide. Correlations between acrylamide, its precursors and crisp colour are described, and the implications of the results for production of potato crisps are discussed.

## Highlights

• Twenty varieties of field-grown potato were stored for 2 months and 6 months at 8 °C.
• Acrylamide contents were measured in crisps prepared from all varieties at both storage times.
• The longer storage period did not affect acrylamide formation significantly.
• Correlations between acrylamide, its precursors and crisp colour are described and discussed.

## 1 Introduction

Cooked potato products, such as crisps, chips (French fries) and oven-cooked potatoes, contribute a substantial proportion of the estimated intake of acrylamide in the adult population of Europe, the other major contributors being coffee and cereal products, in particular bread but also biscuits, crispbreads and breakfast cereals (European Food Safety Authority, 2014). There are considerable differences in dietary preferences across Europe, but crisps account for between 0.6% and 6.6% of adult dietary acrylamide intake and other fried potato products account for between 9.6% and 49%. The percentage contribution of potato products may be even higher for children and adolescents (Dybing et al., 2005), partly because this group drinks less or no coffee. A similar picture has emerged in other parts of the world; Katz, Winter, Buttrey, and Fadel (2012), for example, suggested that 55% of the acrylamide consumed in a typical US diet could be derived from cooked potato products.

Although it has not been established that acrylamide at the levels found in food is harmful to humans, there is a broad consensus based on animal studies that it potentially increases the risk of developing cancer, and in 2011 the Joint FAO/WHO Expert Committee on Food Additives recommended that food manufacturers should make “further efforts on developing and implementing mitigation methods for acrylamide in foods of major importance for dietary exposure” (WHO Technical Report Series No. 959, 2011). A concerted effort by the European snack foods industry in response to this and previous recommendations has led to a significant reduction in acrylamide levels in crisps over the past 10 years (Powers, Mottram, Curtis, & Halford, 2013). Nevertheless, crisp and French fry manufacturers still have to contend with a highly variable raw material and the identification of potato varieties with consistently low concentrations of acrylamide precursors (free asparagine and reducing sugars) in the tubers would make it easier for manufacturers to ensure that acrylamide levels in their products were always low.

Many strategies have been suggested for acrylamide reduction in cooked foods (Taeymans et al., 2004; Vinci, Mestdagh, & De Meulenaer, 2012) and these have been compiled in a ‘Toolbox’ for acrylamide reduction by Food Drink Europe (2014). However, most of these strategies are not applicable to potato crisps and fries, or have an adverse effect on product quality because they affect the Maillard reaction (the heat-induced reaction between free amino acids and reducing sugars), which is the primary mechanism for acrylamide formation but is also crucial for the development of flavour and colour in cooked foods (Mottram, Low, & Elmore, 2006).

A complementary approach is to reduce the concentrations of the precursors for acrylamide formation in the raw material, for example by variety selection (Halford et al., 2012b; Muttucumaru, Powers, Elmore, Mottram, & Halford, 2013; Muttucumaru et al., 2014; Olsson, Svensson, & Roslund, 2004). Raw materials with low precursor concentration would generate less acrylamide in any form of cooking, industrial or domestic, and would reduce the need to adapt processes (Halford et al., 2012a). Hence, potato varieties that are low in acrylamide precursors but give desirable sensory attributes when fried or oven baked are keenly sought.

The aim of this work was: (1) to show that varieties being grown and used for crisp manufacture in the UK are appropriate with regard to their acrylamide-forming potential; (2) to examine the potential of other varieties for crisp manufacture; (3) to provide more data on the effect of tuber storage on acrylamide formation and quality in crisps.

## 2 Materials and methods

### 2.1 Potato samples

Twenty different potato (*Solanum tuberosum*) cultivars grown at the Woburn farm site of Rothamsted Research in Bedfordshire, United Kingdom (Grid reference SP968364; 52°01′06″N, 0°35′30′W; sandy clay loam), in 2011 were analysed in this study. The varieties analysed were Lady Claire, Lady Blanca, Lady Olympia, Lady Rosetta, Daisy, King Edward, Maris Piper, Fontane, Hermes, Markies, Harmony, Pentland Dell, Desiree, Challenger, Ramos, Innovator, Umatilla Russet, Russet Burbank, Saturna and Verdi. Three plots of each variety were grown using a randomised block design; each plot served as a replicate for all subsequent analyses. Uniform application of fertiliser (Nitram (ammonium nitrate; 34.5% N) at 290 kg/ha; triple superphosphate (46% phosphate) at 111 kg/ha; muriate of potash (potassium chloride) at 500 kg/ha) took place immediately prior to planting, in mid-April 2011. Tubers were harvested in September and October 2011, according to whether they were early-, mid- or late-maturing varieties and to when canopy senescence was complete. Plots were irrigated and sprayed as and when deemed necessary by the farm manager. The varieties chosen covered a range of possible food uses, in particular crisps, French fries, and fresh ware (i.e., domestic use, such as mashed, jacket and roast potatoes). Potatoes were all grown at the same site, so that the effect of location on tuber composition was minimised.

Tubers were stored at 8 °C for either 2 or 6 months at the Potato Council Sutton Bridge Crop Storage Research (SBCSR) facility. These conditions were chosen as a suitable compromise for all of the varieties; some varieties used for crisping are stored at higher temperatures while others are not stored for as long as 6 months. Tubers were treated with the anti-sprout agent chlorpropham (CIPC), with applications just after storage commenced and two further applications for the 6-month samples (all applications at 28 mL/tonne of ProLong (50% w/v CIPC in methanol)). Solids contents of tubers were measured when the tubers came out of storage (Tai, Misener, Allaby, & McMillan, 1985).

### 2.2 Crisps

Potatoes from each variety at each storage point (40 treatments) were made into crisps at SBCSR. Three batches of crisps were prepared and analysed for each treatment; each batch corresponded to the contents of a field plot. The procedure used for cooking was typical of that used in the preparation of commercial crisps and gives crisps with an average moisture content of 1.5%.

Tubers were peeled then sliced longitudinally to give slices 0.12–0.15 cm thick. Slices (300 g fresh weight) were washed in cold water for 45 s, stirring continuously and then cooked in 15 L of high oleic sunflower oil for 3 min at a starting temperature of 177 °C, using a Bartlett Yeoman D-11E30 single tank 9 kW electric fryer (Bartlett, Exeter, UK). Crisp samples were allowed to cool, and then heat-sealed in laminated foil. Crisps were stored at −18 °C until analysis. These conditions were chosen as being suitable for all of the varieties but may differ slightly from commercial practice.

### 2.3 Free amino acids and sugars in freeze-dried tubers

Free amino acids and sugars were measured in flour samples prepared from individual freeze-dried tubers (Halford et al., 2012b). Free amino acids (sample size 0.100 ± 0.005 g) were extracted in 10 mL of 0.01 M HCl, derivatised using EZ-Faast (Phenomenex, Torrance, CA) and then analysed by GC–MS. Amino acid concentrations were expressed as mmol/kg dry weight. This method is not suitable for the measurement of arginine. Sugars (sample size 0.100 ± 0.005 g) were extracted in 10 mL of 50% aqueous methanol containing 100 mg/L trehalose and quantified by ion chromatography with pulsed amperometric detection. Sugar concentrations were expressed as mmol/kg dry weight.

### 2.4 Acrylamide analysis

The method of Halford et al. (2012b) was adapted. Potato crisps were ground in a food processor and ground samples (0.500 ± 0.005 g) were weighed into 50-mL Falcon tubes. Samples were extracted with water (40 mL, containing 50 μg/L ^13^C_3_-acrylamide internal standard) at room temperature. After shaking for 20 min, tube and contents were centrifuged at 9000 rpm for 15 min at 15 °C. A discrete fat layer formed on the surface of the sample. Two millilitres were removed from the aqueous layer and passed through a 0.2-μm syringe filter into a 2-mL vial.

Samples were analysed by liquid chromatography-mass spectrometry/mass spectrometry (LC–MS/MS) using an Agilent 1200 high-performance liquid chromatography (HPLC) system connected to a 6410 triple quadrupole mass spectrometer with electrospray ion source in positive ion mode. An isocratic separation was carried out at room temperature using a 100 × 3.0 mm Hypercarb column with a 10 × 3.0 mm Hypercarb pre-column (both 5 μm particle size; Thermo Fisher, Waltham, MA). The mobile phase was 0.1% aqueous formic acid at a flow rate of 0.3 mL/min. Injection volume was 25 μL. The transitions *m*/*z* 72 → 55 and *m*/*z* 72 → 27 were measured for acrylamide and the transition *m*/*z* 75 → 58 was measured for ^13^C_3_-acrylamide. Concentrations of acrylamide in crisps were expressed as μg/kg fresh weight.

### 2.5 Crisp colour measurement

Fried crisp samples were first graded in a light cabinet to remove any surface defects, such as greening, bruising and excessively dark fry colour. Removal of defects often resulted in insufficient sample to present to the Hunter Lab, so *Lab* values could not be obtained for some samples. The sample remaining was placed in a shallow dish, and the surface was crushed flat before presenting to the viewing port of a Hunter DP-9000 colorimeter (Hunter Associates Laboratory Inc., Reston, VA). The sample dish was turned through approximately 120° before taking a second reading, and again for a third reading. Samples did not leave the dish between readings. The three readings were averaged, to give one value each of *L*, *a* and *b* per sample.

### 2.6 Statistical analysis

XLStat 2012 (Addinsoft, Paris, France) was used to perform two-way analysis of variance on the acrylamide, colour, amino acid and sugars data (*p* = 0.05). Microsoft Excel 2010 was used for student’s *t*-test (significance at *p* = 0.05) and regression plots.

## 3 Results

### 3.1 Tuber properties

Twenty varieties of potatoes grown at Woburn, Bedfordshire, UK in 2011 were stored for 2 and 6 months, and then made into crisps. The varieties studied covered a range of uses, i.e., crisp manufacture, chip/French fry manufacture and fresh ware, i.e., domestic use. These varieties made up around half of the total acreage of potatoes grown in the UK in 2013 (Table 1). The total acreage includes all types of potato, such as salad potatoes and tubers grown for seed. Tables 2–4 are all subdivided into three sections based on the culinary use information provided in Table 1; i.e., crisping varieties are listed first, followed by French fry varieties then boiling varieties.

Acrylamide and *Lab* colour were measured in crisps made from all varieties, after storage at both 2 and 6 months. It is important to consider storage when analysing potato crisps because crisp shelf life is only 1–2 months. Therefore, in order for there to be a continuous supply, crisps must be manufactured throughout the year, which means the use of potatoes stored for up to 10 months (Lisińska & Leszczyński, 1989). Free amino acids and sugars were also measured in freeze-dried tubers of all varieties stored under the same conditions, in order to increase our understanding of how the concentration of acrylamide precursors in tubers affects acrylamide formation in crisps.

The average dry matter contents for each variety are shown in Table 1. Dry matter content of tubers varied significantly between varieties but was unaffected by storage (data not shown). Verdi had significantly higher dry matter content than all the other varieties (28.3%; *p* < 0.05), while the dry matter contents of Harmony (17.7%) and Lady Blanca (21.4%) were significantly lower than those of the other varieties and different from each other (*p* < 0.05). The dry matter contents of the other 17 varieties were all between 23.5% and 26.0%. All crisping varieties were greater than 25% dry matter, with the exception of Markies (a variety traditionally regarded as French fry but recently also used for crisping), which was 24.3%, while Russet Burbank was the only non-crisping variety with a dry matter content of greater than 25% (25.2%).

### 3.2 Free amino acids and asparagine

Of the twenty amino acids measured, only glutamic acid was not affected by variety, while only γ-aminobutyric acid (GABA), glutamine and histidine were not affected by storage. Although the large number of significant interactions suggested that variety and storage effects were not consistent for particular amino acids, both alanine and proline doubled in concentration as a result of storage, while glutamic acid halved in concentration (Supplementary data: Table S1).

Table 3 shows the effect of storage on asparagine concentrations in all twenty varieties. Five varieties show a significant decrease in asparagine with storage; in particular, Verdi, which is relatively low in asparagine at 2 months anyway, reduces in asparagine by nearly 60% after 6 months. Knutsen et al. (2009) also showed that asparagine did not change in Saturna stored for 6 months at 8 °C.

In general, our group and other workers have found asparagine to be the free amino acid found at the highest levels in potatoes (Halford et al., 2012b; Matsuura-Endo et al., 2006; Muttucumaru et al., 2013, 2014; Viklund, Olsson, Sjoholm, & Skog, 2008a), but for Verdi in this experiment both glutamic acid and aspartic acid were present at higher levels than asparagine. The same effect was observed for the varieties Lady Claire and Fontane; for Challenger and Daisy aspartic acid was at higher levels than asparagine, while glutamic acid was at lower levels. Similar results were obtained for a range of varieties by Zhu, Cai, Ke, and Corke (2010).

The relative concentration of asparagine compared to the total free amino acid concentration is also shown in Table 2. No obvious trend was observed between potato culinary use and asparagine concentration, relative asparagine concentration or total free amino acid content.

### 3.3 Sugars

The data revealed massive differences in reducing sugar contents, from 3.72 mmol/kg dry weight in unstored Verdi, all the way up to 572 mmol/kg dry weight in stored Harmony. Total reducing sugars increased in five varieties as storage time increased; those varieties were Daisy, Innovator, Lady Rosetta, Markies and Ramos. The biggest increase was in Lady Rosetta, where the mean total reducing sugars at 6 months was 2.74 times higher than at 2 months (Table 3). In Lady Rosetta and Innovator sucrose also increased on storage, while in Daisy and Ramos, sucrose decreased; in Markies sucrose was unaffected.

A previous study on nine varieties of potato from commercial suppliers revealed significant interactions between varieties nested within type (French fry and crisping) and storage time for most free amino acids, glucose, fructose, and acrylamide formation (Halford et al., 2012b), but the potatoes were relatively stable until late storage (>6 months) when they approached and passed their recommended storage window. This led to the recommendation that potatoes that have been stored beyond their recommended storage window should not be used for processes in which acrylamide might form. However, another study, on 13 potato varieties grown together at the same site as the one used for this study but in the previous season and stored for 6 months at 9 °C at the same facility did show a significant variety-dependent impact of storage on sugars, free amino acids and acrylamide-forming potential (Muttucumaru et al., 2014).

Amrein et al. (2004) showed a large decrease in reducing sugars in three varieties stored at 8 °C for 6 months, although storage management information was not provided, while Kyriacou, Ioannides, Gerasopoulos, and Siomos (2009) observed no obvious trends in four different varieties stored at 8.5 °C. A review by Kumar, Singh, and Kumar (2004) highlights the large number of interacting factors which influence sugar content and composition during storage.

### 3.4 Acrylamide

The acrylamide contents for crisps made from the 20 varieties are shown in Table 4. The effect of variety on acrylamide formation was highly significant (*p* < 0.001). Concentrations ranged from just over 100 μg/kg in variety Verdi to 5.5 mg/kg in Pentland Dell, i.e., a factor of 50 between the lowest and highest values. Of the five varieties that are suggested by the Potato Council as suitable for making crisps, that is Hermes, Lady Claire, Lady Rosetta, Saturna and Verdi, all except Hermes produced crisps that contained levels of acrylamide that were towards the lower end of the range and below the 1000 μg/kg indicative level set by the European Commission (European Commission, 2011). This was consistent with previous studies in which Lady Claire, Lady Rosetta, Saturna and Verdi have all performed well with respect to acrylamide formation (Halford et al., 2012a; Halford et al., 2012b; Muttucumaru et al., 2013, 2014). Markies has generally been regarded as a French fry variety but is being used increasingly for crisping as well, particularly after prolonged storage, when its excellent storage stability makes it a better option than some dedicated crisping varieties (Halford et al., 2012b; Muttucumaru et al., 2014). It performed well in this study, as did another French fry variety, Fontane. The other varieties that are widely used domestically in the UK, such as Maris Piper, Desiree, and King Edward, were towards the higher end of the range, as was the boiling type Harmony (http://varieties.potato.org.uk/menu.php). Russet Burbank and Umatilla Russet, both popular French fry varieties for the North American fast food industry, were also towards the higher end of the range, and considerably higher than some other French fry varieties, while the worst French fry variety with respect to acrylamide formation was Pentland Dell. These varieties have been shown to have high acrylamide-forming potential in previous studies as well (Halford et al., 2012b; Muttucumaru et al., 2013, 2014). However, it must be borne in mind that the crisps were cooked for a fixed time (3 min) in a fryer at 177 °C. Varieties with high acrylamide content were generally much darker (see Section 3.5) and in practice they would be cooked to a much paler end point in order to be acceptable visually. The crisp industry has traditionally selected varieties that would give a pale colour as well as the desired final texture and moisture content. In general these varieties have lower sugar content.

In contrast with previous studies (Halford et al., 2012b; Muttucumaru et al., 2014) analysis of variance did not show a significant effect of storage on acrylamide formation overall. Student’s *t*-test did show that acrylamide in the variety Innovator increased significantly upon storage (*p* < 0.05), doubling in value, but Saturna showed a small but significant decrease in acrylamide on storage (*p* < 0.001). Hermes, Lady Rosetta, Challenger, Daisy and Harmony all showed increases in acrylamide after the longer storage period but the differences were not statistically significant.

Saturna, Lady Claire and Lady Rosetta are varieties commonly used for crisp manufacture in the UK (Table 1), while Markies is becoming increasingly popular as both a French fry and crisping variety. Fontane and Lady Claire (along with Innovator) were assessed by Swisspatat shortly after their introduction as new varieties. Fontane was suggested as being excellent for manufacture of chips, crisps and rösti, while Lady Claire with its very low reducing sugar content was deemed ideal for crisp manufacture (Hebeisen, Ballmer, Reust, & Bertossa, 2003).

Krause et al. (2006) stated that Verdi was an optimum variety for the manufacture of crisps because even after storage at 4 °C for 4 months, tuber reducing sugar levels were low. However, Pedreschi, Mariotti, Granby, and Risum (2011) prepared crisps from Verdi potatoes stored at 8 °C, which contained around 2000 μg/kg acrylamide. Hermes is widely grown in the UK as a crisping variety (Table 1), yet in this experiment both unstored and stored potatoes gave crisps that were higher than the other crisping varieties.

Halford et al. (2012b) measured acrylamide in crisps made from tubers stored for up to 10 months. The nine varieties that they studied were also studied in this trial. They showed that Saturna, Lady Claire and Lady Rosetta gave crisps that were consistently lower than 1000 μg/kg for acrylamide during the first months of storage but rose above the indicative level in Lady Rosetta, which has a relatively short optimum storage window, after the 6th month of storage; Markies crisps, on the other hand, were lower for acrylamide in the later months of storage as its reducing sugar levels declined, while Hermes crisps initially gave low acrylamide values, although a value of 1533 μg/kg was obtained after 6 months, followed by a considerable increase during the last 3 months of storage. Maris Piper was below 1000 μg acrylamide/kg crisps at 2 months but not at 6 months, while Daisy, King Edward, and Pentland Dell gave crisps that were consistently above 1000 μg/kg.

**Fig. 1:**
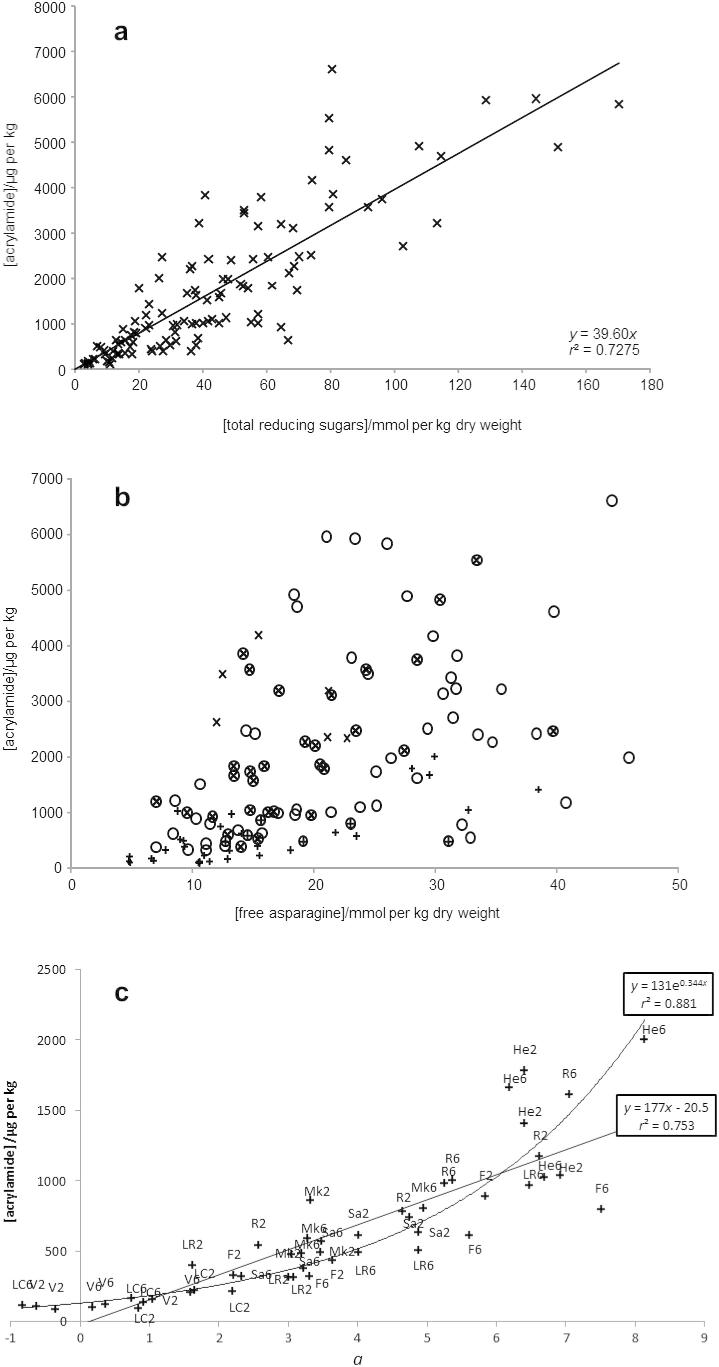
Relationships between acrylamide content (μg/kg) and (a) reducing sugars (mmol/kg dry weight), (b) free asparagine (mmol/kg dry weight), and (c) colour (*a*) for potato crisps made from UK-grown potato varieties stored for 2 and 6 months at 8 °C. In (a) data for variety Harmony are not included. (b) Shows varieties suitable for crisping (+), French fries (○), fresh ware (×), French fries and crisps (⊕), French fries and fresh ware (⊗). Varieties in (c) are Fontane (F), Hermes (He), Lady Claire (LC), Lady Rosetta (LR), Markies (Mk), Ramos (R), Saturna (Sa), and Verdi (V).

Many papers have highlighted the correlation between reducing sugar content in the tuber and acrylamide formation in crisps (Matsuura-Endo et al., 2006; Ohara-Takada et al., 2005; Viklund, Olsson, Sjoholm, & Skog, 2008b; Viklund et al., 2008a). In the present study, when acrylamide content (in μg/kg) was plotted against total reducing sugars (in mmol/kg dry weight) for all twenty varieties, Harmony was an obvious outlier with a relatively low acrylamide formation relative to its reducing sugar content. When the values for Harmony were removed from the plot, a linear relationship was obtained (*r*^2^ = 0.728; Fig. 1a). Subsets based on culinary use (crisps/chips, French fries or fresh ware) showed a weaker correlation between reducing sugars and acrylamide formation (data not shown).

This effect shown by Harmony is of interest. Acrylamide formation in potato products is normally proportional to reducing sugar content because reducing sugars are usually present in tubers at low levels compared to free amino acids. Even allowing for the absence of arginine in the measured amino acid data, reducing sugars in Harmony were so high that, on a molar basis, they exceeded the total free amino acid content by 4 to 7-fold. Although reducing sugars were also higher than total free amino acids in Pentland Dell, they were only up to 1.5 times higher, whereas in the other varieties free amino acids were in excess. As total amino acids and asparagine content in Pentland Dell and Harmony were similar (Table 3), it might be expected that their acrylamide contents would be similar. The significantly lower levels of acrylamide in Harmony relative to Pentland Dell (*p* < 0.001; Student’s *t*-test) are therefore surprising and difficult to explain.

When acrylamide content (*y* in μg/kg) was plotted against asparagine content (*x* in mmol/kg dry weight) a positive correlation was observed *y* = 89.5*x* (*r*^2^ = 0.27; *p* < 0.001). For crisping varieties the correlation was stronger still: *y* = 37.8*x* (*r*^2^ = 0.51; *p* < 0.001). Fig. 1b shows how acrylamide formed varied with free asparagine content for all 120 samples.

### 3.5 Crisp colour

Visual inspection of the crisps indicated that those which were highest in acrylamide were the brownest, while those that were low in acrylamide were relatively pale. All crisps were assessed for *Lab* colour but many of the varieties that are not usually used in crisp manufacture could not be analysed because they were uneven in colour or contained too many surface defects. In fact, *Lab* values were only obtained for 8 varieties: those six varieties listed in Table 1 as being suitable for crisping, plus Ramos and Fontane.

There was a highly significant effect of variety on *L*, *a* and *b* (*p* < 0.0001), with crisps made from variety Verdi being the palest and greenest (high *L*, low *a*) and those made from Hermes being the darkest and reddest (low *L*, high *a*). In addition, *L* showed a slight but highly significant decrease with storage (*p* < 0.0001). In particular, *L*-values for Lady Rosetta and Fontane decreased significantly after 6-months storage (*p* < 0.05), resulting in darker crisps.

There was a good linear correlation between acrylamide content and *a*-value (*y* = 177*x* − 20.5; *r*^2^ = 0.753; Fig. 1c), while a weaker inverse correlation was observed between *L* and acrylamide (*y* = −134*x* + 8820; *r*^2^ = 0.6882), results being similar to those reported by other workers (Halford et al., 2012b; Pedreschi, Kaack, & Granby, 2006; Serpen & Gökmen, 2009). However, three readings for *a* value were negative, resulting in negative acrylamide values using the proposed equation. A higher correlation between acrylamide and *a* value was obtained using an exponential relationship (*y* = 131e^0.344^*^x^*; *r*^2^ = 0.881); this was particularly noticeable at higher acrylamide concentrations. A similar correlation was observed in French fries by Mestdagh et al. (2008).

## 4 Discussion

This work has shown within a controlled experiment that there are very large varietal differences in the water-soluble components of potato that are responsible for the quality attributes of cooked potato, including acrylamide. Total free amino acids varied over the range 73 to 137 mmol/kg dry weight with asparagine varying from 14% to 29% of the total free amino acids. Sugars showed much greater variation, ranging from 3.7 to 520 mmol/kg.

Between 2002 and 2011, modification of cooking practices and improved management of tuber storage resulted in a 53% decrease in acrylamide levels in potato crisps produced in Europe (Powers et al., 2013). Even so, the need for a further reduction in acrylamide levels in crisps remains, in order for manufacturers to keep up with an evolving regulatory situation. The Food Drink Europe Acrylamide Toolbox (2014) recommends the use of the ALARA (As Low As Reasonably Achievable) concept, where food manufacturers use the mitigation strategies described in the Toolbox to reduce acrylamide levels in their products.

It is clear from published literature that levels of reducing sugars in tubers are affected by variety, storage (temperature, use of sprout inhibitors, atmosphere), and growing conditions (rainfall, temperature and mineral content) (Kumar et al., 2004), with effects often being variety-dependent (Muttucumaru et al., 2013, 2014). Although reducing sugar content is well correlated with acrylamide formation, predictions of acrylamide formation in crisps are difficult to make with confidence, unless a variety is chosen that has been shown to produce crisps consistently with levels of acrylamide below the indicative value of 1000 μg/kg set by the European Commission (European Commission, 2011). The correlation between *Lab* values and acrylamide content may be a useful means for a manufacturer to have confidence that it is producing crisps below the indicative value. Based on the two curves plotted in Fig. 1c, a value for *a* of greater than 5 for a crisp sample may be a cause for concern.

Of the crisping varieties regularly used in the United Kingdom, Lady Claire performed consistently well, with all 6 crisp samples containing below 230 μg/kg acrylamide and with a mean value of 158 μg/kg. Saturna, Markies and Lady Rosetta gave similar mean values of 542, 616 and 498 μg/kg, respectively, but highest values were approaching the indicative value, particularly a Lady Rosetta sample that gave a value of 965 μg/kg. Of course, this work was carried out with relatively small samples compared with those used by crisp manufacturers, and it is likely that batches of tubers used commercially would give acrylamide values showing less variability than the values observed in this experiment. A dry matter content of greater than 21% is suggested for crisping varieties (Knight, 1991), although in our work only Harmony gave tubers with dry matter content below 21%; dry matter content showed a small but highly significant negative correlation with acrylamide content (*r*^2^ = 0.163; *p* ⩽ 0.001).

Of possible potential interest to crisp manufacturers are the varieties Verdi and Fontane, with mean values of 131 and 563 μg/kg, respectively. However, the variety Hermes, which was introduced in 1973 (www.europotato.org/menu.php) and has been popular for a number of years consistently contained more than 1000 μg/kg acrylamide, with one sample stored for 6 months containing 2000 μg/kg; these results are similar to those obtained in a previous study (Halford et al., 2012b). Plantings of the crisping variety Hermes in Great Britain have decreased since 2011 (Table 5), with varieties introduced since this trial took place, such as VR808, Taurus and Shelford, becoming increasingly popular.

Acrylamide-forming potential is of key importance when choosing a variety for the manufacture of crisps. However, acrylamide, although it does not contribute to crisp flavour, is formed in the Maillard reaction in parallel with flavour (Mottram et al., 2006). As a result, crisps low in acrylamide may have a relatively bland flavour. In addition, a crisp that is low in acrylamide may also be undesirable in terms of texture. If tuber dry matter content is above 25%, crisps may be perceived as being too hard (Lisińska, 1989). Verdi, with a dry matter content of 28% (Table 1), could suffer from this problem. Both flavour and crispness are attributes that are positively correlated with overall sensory quality (Jaworska & Hoffmann, 2008).

For most of the varieties examined in this work, there was no significant difference in acrylamide levels in crisps made from potatoes after 6 months of storage compared with potatoes processed after only 2 months of storage (*p* > 0.05). However, previous studies have shown a significant effect of storage (Halford et al., 2012b; Muttucumaru et al., 2014), particularly at longer storage times. When Halford et al. (2012b) stored potatoes for up to 9 months, it could be seen that the largest increases in acrylamide levels occurred between months 6 and 9, while increases in acrylamide formation in crisps made from potatoes stored for up to 6 months were small or non-existent. We continue to recommend that potatoes be used within their optimal storage window.

## 5 Conclusions

Although many papers have been published demonstrating how acrylamide can be reduced in cooked potato products by modification of process, variety selection is of great importance. In this paper we have shown that varieties currently used in the UK for crisp manufacture are generally appropriate for producing crisps with acrylamide content less than the indicative value of 1000 μg/kg that has been set by the European Commission (2011), although the use of Hermes for crisp manufacture may need to be reconsidered. Providing that they are of appropriate sensory quality, Fontane and Verdi may be newer varieties suitable for producing low-acrylamide crisps.

## Figures and Tables

**Table 1:** Studied potato varieties with culinary uses, dry matter content and production information.

Variety	Culinary use^a^	Dry matter content (%)^b^	Planted area (ha)^c^	Percentage of planted area
Hermes	Crisps	25.8	3457	2.82
Lady Claire	Crisps	25.8	1075	0.88
Lady Rosetta	Crisps	25.3	7101	5.80
Saturna	Crisps	26.0	1081	0.88
Verdi	Crisps	28.3	0	
Challenger	French fries	24.0	978	0.80
Daisy	French fries; fresh ware	24.2	239	0.20
Desiree	French fries; fresh ware	23.7	2528	2.06
Fontane	French fries	24.8	1921	1.57
Innovator	French fries; fresh ware	24.1	1556	1.27
King Edward	French fries; fresh ware	24.1	27.7	2.24
Lady Blanca	French fries	21.4	0	
Lady Olympia	French fries	24.0	1	0.00
Maris Piper	French fries; fresh ware	24.1	18,213	14.87
Markies	French fries; crisps	24.3	6988	5.71
Pentland Dell	French fries	24.9	2998	2.45
Ramos	French fries	23.5	1194	0.98
Russet Burbank	French fries	25.2	1787	1.46
Umatilla Russet	French fries	24.2	195	0.16
Harmony	Boil; fresh ware	17.7	3807	3.11

a Culinary use data obtained from British Potato Variety Database (www.varieties.potato.org.uk/menu.php), European Cultivated Potato Database (www.europotato.org/menu.php), and Netherlands Potato Consultative Foundation (www.potato.nl).

b Mean value for tubers stored for 2 and 6 months (*n* = 6).

c Data are for potatoes planted in 2013 in the United Kingdom (excluding Northern Ireland); data obtained from Hannah Goodwin, AHDB Potato Council (personal communication).

**Table 2:** Free asparagine, total free amino acids and relative amount of free asparagine (mmol/kg dry weight) in 20 varieties of potatoes grown in the UK (mean ± standard deviation, *n* = 3).

variety	2-Months storage			6-Months storage			*p*^a^
	Free asparagine	Total free amino acids^b^	Asparagine/total (%)	Free asparagine	Total free amino acids	Asparagine/total (%)	
Hermes	33.1 ± 5.21	113 ± 8.17	29.4	22.8 ± 12.1	88.3 ± 23.8	25.8	NS
Lady Claire	12.4 ± 2.77	76.1 ± 10.1	16.2	8.0 ± 2.25	60.0 ± 5.85	13.4	NS
Lady Rosetta	15.5 ± 2.51	87.1 ± 10.4	17.8	10.5 ± 2.34	63.9 ± 6.53	16.5	NS
Saturna	16.1 ± 5.02	73.5 ± 5.99	21.9	13.6 ± 8.65	59.5 ± 19.5	22.9	NS
Verdi	11.6 ± 1.20	66.4 ± 11.4	17.6	4.8 ± 0.04	38.7 ± 1.35	12.5	^∗∗∗^

Challenger	18.3 ± 5.56	88.2 ± 14.6	20.8	8.8 ± 1.85	62.1 ± 6.83	14.1	^∗^
Daisy	14.1 ± 1.22	98.7 ± 11.3	14.3	11.6 ± 1.94	81.8 ± 8.07	14.1	NS
Desiree	30.5 ± 9.14	132 ± 20.0	23.2	26.4 ± 8.36	105 ± 26.3	25.1	NS
Fontane	10.4 ± 0.76	72.6 ± 14.5	14.3	10.4 ± 1.68	70.5 ± 3.77	14.7	NS
Innovator	18.7 ± 3.18	89.1 ± 3.74	21.0	17.8 ± 5.69	89.7 ± 10.1	19.8	NS
King Edward	16.4 ± 3.22	118 ± 21.3	13.9	13.7 ± 6.20	103 ± 25.1	13.3	NS
Lady Blanca	37.5 ± 7.65	131 ± 23.1	28.6	35.6 ± 4.18	116 ± 7.76	30.8	NS
Lady Olympia	22.3 ± 3.37	99.4 ± 14.2	22.5	18.7 ± 5.62	94.3 ± 3.99	19.9	NS
Maris Piper	22.6 ± 5.43	113 ± 19.6	19.9	17.2 ± 2.96	81.6 ± 16.5	21.1	NS
Markies	22.0 ± 8.10	88.6 ± 13.2	24.8	16.8 ± 5.50	65.5 ± 13.3	25.6	NS
Pentland Dell	25.7 ± 2.19	122 ± 3.21	21.1	19.4 ± 1.48	88.0 ± 8.77	22.0	^∗^
Ramos	35.3 ± 4.74	137 ± 15.5	25.8	22.4 ± 5.79	84.0 ± 11.9	26.6	^∗^
Russet Burbank	37.5 ± 7.48	145 ± 24.8	26.0	29.8 ± 3.00	102 ± 8.51	29.3	NS
Umatilla Russet	29.6 ± 4.62	136 ± 27.9	21.7	19.4 ± 9.07	86.3 ± 28.7	22.4	NS

Harmony	21.7 ± 0.88	96.4 ± 1.22	22.5	13.3 ± 1.86	81.9 ± 4.52	16.3	^∗∗^

a Significant effect of storage time on asparagine concentration (^∗^*p* < 0.05, ^∗∗^*p* < 0.01 and ^∗∗∗^*p* < 0.001; NS, not significant).

b Excludes arginine.

**Table 3:** Total reducing sugar levels (mmol/kg dry weight) in 20 varieties of potatoes grown in the UK (mean ± standard deviation, *n* = 3).

Variety	2-Months storage	6-Months storage	*p*^a^
Hermes	21.0 ± 2.28	42.6 ± 14.56	NS
Lady Claire	9.72 ± 3.22	8.67 ± 3.22	NS
Lady Rosetta	10.8 ± 2.39	29.7 ± 2.67	^∗∗∗^
Saturna	15.6 ± 2.39	13.6 ± 1.28	NS
Verdi	3.72 ± 0.72	5.17 ± 1.22	NS

Challenger	31.5 ± 10.4	35.2 ± 7.00	NS
Daisy	31.5 ± 7.00	58.4 ± 6.06	^∗∗^
Desiree	69.7 ± 9.61	80.2 ± 15.9	NS
Fontane	20.8 ± 4.39	38.9 ± 25.1	NS
Innovator	45.3 ± 9.06	84.22 ± 6.72	^∗∗^
King Edward	42.8 ± 16.8	65.33 ± 6.67	NS
Lady Blanca	70.2 ± 12.9	101 ± 14.3	NS
Lady Olympia	46.1 ± 11.0	39.4 ± 9.67	NS
Maris Piper	60.9 ± 13.6	42.28 ± 8.94	NS
Markies	10.3 ± 4.39	18.3 ± 0.61	^∗^
Pentland Dell	150 ± 20.8	122 ± 19.4	NS
Ramos	18.6 ± 4.50	37.61 ± 0.72	^∗∗^
Russet Burbank	41.9 ± 5.67	46.3 ± 7.17	NS
Umatilla Russet	53.3 ± 4.22	46.9 ± 24.6	NS

Harmony	452 ± 55.7	572 ± 66.4	NS

a Significant effect of storage time on reducing sugar concentration (^∗^*p* < 0.05, ^∗∗^*p* < 0.01 and ^∗∗∗^*p* < 0.001; NS, not significant).

**Table 4:** Acrylamide levels (μg/kg) in potato crisps made from 20 varieties of potatoes grown in the UK (mean ± standard deviation, *n* = 3).

Variety	2-Months storage	6-Months storage	*p*^a^
Hermes	1410 ± 372	1560 ± 499	NS
Lady Claire	178 ± 71	138 ± 27	NS
Lady Rosetta	340 ± 47	655 ± 271	NS
Saturna	663 ± 69	422 ± 132	^∗^

Verdi	119 ± 36	142 ± 55	NS
Challenger	812 ± 371	1030 ± 586	NS
Daisy	496 ± 114	1240 ± 504	NS
Desiree	3460 ± 1220	4150 ± 1226	NS
Fontane	550 ± 297	576 ± 241	NS
Innovator	1850 ± 318	3660 ± 168	^∗∗∗^
King Edward	1480 ± 468	1730 ± 531	NS
Lady Blanca	3840 ± 2386	3500 ± 984	NS
Lady Olympia	2190 ± 1424	1390 ± 924	NS
Maris Piper	1860 ± 759	1300 ± 482	NS
Markies	609 ± 219	624 ± 164	NS
Pentland Dell	5540 ± 572	5180 ± 674	NS
Ramos	833 ± 319	1200 ± 357	NS
Russet Burbank	2690 ± 992	2870 ± 785	NS
Umatilla Russet	3000 ± 559	2430 ± 1743	NS

Harmony	2620 ± 493	3430 ± 778	NS

a Significant effect of storage time on acrylamide concentration (^∗^*p* < 0.05 and ^∗∗∗^*p* < 0.001; NS, not significant).

**Table 5:** Planting areas (in hectares) of major crisping varieties in the United Kingdom (excluding Northern Ireland) from 2007 to 2014 (data from Hannah Goodwin, AHDB Potato Council; personal communication).

Variety	Planting year							
	2007	2008	2009	2010	2011	2012	2013	2014
Markies	3507	4012	6257	7132	7415	7520	6988	7527
Lady Rosetta	6035	5664	6922	6831	6764	6525	7101	5322
Hermes	4011	4061	4021	4107	4778	4580	3457	2724
Agria	254	311	433	766	1048	1169	2054	1830
VR808	–	–	–	–	67	161	907	1475
Lady Claire	606	626	633	1025	937	1076	1075	916
Taurus	–	–	–	–	–	114	602	774
Saturna	4905	4115	3539	3578	3241	2449	1081	719
Shelford	–	–	–	–	–	–	78	607

## Appendix A Supplementary data

Supplementary Table S1 Free amino acid concentrations in 20 varieties of UK-grown potatoes after 2 and 6 months of storage (mean ± standard deviation, *n* = 3).
