# Supplementary material for: Acrylamide in potato crisps prepared from 20 UK-grown varieties: Effects of variety and tuber storage time
Source: Food Chem. 2015 Sep 1;182:1–8. doi: 10.1016/j.foodchem.2015.02.103 (PMC4396699; doi:10.1016/j.foodchem.2015.02.103)
Supplement: Supplementary Table S1 — Free amino acid concentrations in 20 varieties of UK-grown potatoes after 2 and 6 months of storage (mean ± standard deviation, n = 3). [file mmc1.docx]

Table S1. Free amino acid concentrations in 20 varieties of UK-grown potatoes after 2 and 6 months of storage (mean ± standard deviation, *n* = 3)

| variety | storage time (months) | Mean amino acid concentration (mmol per kg dry weight) | | | | | | | | | | | | | | | | | | | | |
| --- | --- | --- | --- | --- | --- | --- | --- | --- | --- | --- | --- | --- | --- | --- | --- | --- | --- | --- | --- | --- | --- | --- |
|  |  | Ala | Gly | Val | Leu | Ile | Thr | Ser | GABA | Pro | Asn | Asp | Met | Glu | Phe | Gln | Orn | Lys | His | Tyr | Try | total |
| *crisping varieties* |  |  |  |  |  |  |  |  |  |  |  |  |  |  |  |  |  |  |  |  |  |  |
| Hermes | 2 | 3.45 ± 0.20 | 0.62 ± 0.07 | 4.03 ± 0.12 | 0.81 ± 0.06 | 1.38 ± 0.09 | 1.26 ± 0.05 | 2.65 ± 0.42 | 9.20 ± 4.80 | 0.99 ± 0.11 | 33.1 ± 5.21 | 22.7 ± 1.88 | 0.79 ± 0.09 | 18.9 ± 5.81 | 1.66 ± 0.15 | 5.34 ± 1.08 | 0.03 ± 0.03 | 2.22 ± 0.36 | 0.84 ± 0.16 | 2.23 ± 0.26 | 0.38 ± 0.05 | 113 ± 8.17 |
|  | 6 | 7.05 ± 4.24 | 0.64 ± 0.09 | 3.81 ± 0.53 | 0.83 ± 0.07 | 1.00 ± 0.05 | 1.13 ± 0.11 | 2.29 ± 0.76 | 10.9 ± 6.29 | 2.47 ± 0.21 | 22.8 ± 12.1 | 12.7 ± 5.27 | 0.26 ± 0.09 | 7.28 ± 2.04 | 1.12 ± 0.21 | 9.47 ± 2.88 | 0.05 ± 0.02 | 1.85 ± 0.08 | 1.04 ± 0.12 | 1.45 ± 0.05 | 0.22 ± 0.04 | 88.3 ± 23.8 |
| Lady Claire | 2 | 2.02 ± 0.23 | 0.43 ± 0.04 | 2.46 ± 0.42 | 0.36 ± 0.07 | 0.73 ± 0.11 | 0.82 ± 0.15 | 1.55 ± 0.28 | 3.62 ± 3.20 | 0.54 ± 0.10 | 12.4 ± 2.77 | 23.9 ± 0.71 | 0.49 ± 0.04 | 18.8 ± 1.42 | 0.99 ± 0.13 | 4.00 ± 1.00 | 0.01 ± 0.02 | 1.05 ± 0.17 | 0.68 ± 0.39 | 1.01 ± 0.20 | 0.35 ± 0.08 | 76.1 ± 10.1 |
|  | 6 | 5.82 ± 1.66 | 0.36 ± 0.04 | 2.57 ± 0.18 | 0.71 ± 0.03 | 0.78 ± 0.02 | 0.62 ± 0.10 | 1.50 ± 0.16 | 5.25 ± 0.37 | 0.82 ± 0.04 | 8.04 ± 2.25 | 11.0 ± 2.96 | 0.38 ± 0.07 | 15.7 ± 2.47 | 0.82 ± 0.10 | 2.63 ± 0.19 | 0.03 ± 0.03 | 1.19 ± 0.13 | 0.56 ± 0.25 | 0.90 ± 0.12 | 0.23 ± 0.04 | 60.0 ± 5.85 |
| Lady Rosetta | 2 | 1.77 ± 0.27 | 0.53 ± 0.15 | 3.42 ± 0.71 | 0.69 ± 0.11 | 1.26 ± 0.30 | 1.12 ± 0.44 | 2.20 ± 0.62 | 6.46 ± 1.12 | 1.17 ± 0.18 | 15.5 ± 2.51 | 18.5 ± 3.39 | 0.54 ± 0.10 | 20.6 ± 7.20 | 1.40 ± 0.20 | 4.26 ± 2.64 | 0.28 ± 0.11 | 4.37 ± 2.49 | 1.41 ± 0.71 | 1.12 ± 0.25 | 0.44 ± 0.17 | 87.1 ± 10.4 |
|  | 6 | 2.80 ± 1.04 | 0.45 ± 0.07 | 3.46 ± 0.28 | 0.81 ± 0.09 | 0.98 ± 0.05 | 0.73 ± 0.13 | 1.78 ± 0.27 | 7.02 ± 2.57 | 2.42 ± 0.17 | 10.5 ± 2.34 | 15.8 ± 4.62 | 0.51 ± 0.03 | 5.98 ± 1.52 | 2.04 ± 0.02 | 4.73 ± 0.82 | 0.12 ± 0.04 | 1.93 ± 0.06 | 0.91 ± 0.24 | 0.75 ± 0.12 | 0.17 ± 0.02 | 63.9 ± 6.53 |
| Saturna | 2 | 1.31 ± 0.10 | 0.39 ± 0.04 | 2.64 ± 0.06 | 0.75 ± 0.02 | 1.18 ± 0.03 | 0.63 ± 0.08 | 1.48 ± 0.17 | 5.81 ± 1.37 | 0.61 ± 0.04 | 16.1 ± 5.02 | 21.2 ± 1.11 | 0.40 ± 0.17 | 11.2 ± 4.31 | 1.03 ± 0.07 | 2.85 ± 0.42 | 0.07 ± 0.03 | 3.00 ± 1.14 | 0.92 ± 0.35 | 1.44 ± 0.05 | 0.43 ± 0.04 | 73.5 ± 5.99 |
|  | 6 | 5.54 ± 3.53 | 0.34 ± 0.07 | 2.52 ± 0.75 | 0.83 ± 0.16 | 1.04 ± 0.22 | 0.52 ± 0.19 | 1.23 ± 0.24 | 4.39 ± 2.69 | 1.56 ± 0.35 | 13.6 ± 8.65 | 9.80 ± 5.64 | 0.44 ± 0.17 | 9.78 ± 5.20 | 0.91 ± 0.31 | 3.53 ± 0.84 | 0.03 ± 0.03 | 1.52 ± 0.50 | 0.47 ± 0.10 | 1.14 ± 0.55 | 0.27 ± 0.12 | 59.5 ± 19.5 |
| Verdi | 2 | 1.12 ± 0.06 | 0.33 ± 0.11 | 1.57 ± 0.25 | 0.36 ± 0.06 | 0.72 ± 0.11 | 0.55 ± 0.18 | 1.41 ± 0.33 | 3.46 ± 2.19 | 0.85 ± 0.17 | 11.7 ± 1.20 | 18.4 ± 4.14 | 0.38 ± 0.12 | 17.4 ± 3.03 | 0.88 ± 0.21 | 3.14 ± 1.49 | 0.03 ± 0.03 | 2.50 ± 1.54 | 0.72 ± 0.42 | 0.63 ± 0.12 | 0.27 ± 0.10 | 66.4 ± 11.4 |
|  | 6 | 2.15 ± 1.17 | 0.38 ± 0.11 | 1.13 ± 0.10 | 0.38 ± 0.04 | 0.73 ± 0.17 | 0.27 ± 0.03 | 0.82 ± 0.21 | 2.16 ± 0.25 | 1.66 ± 0.04 | 4.84 ± 0.04 | 10.2 ± 5.17 | 0.21 ± 0.05 | 9.37 ± 3.75 | 0.75 ± 0.13 | 2.25 ± 0.45 | 0.01 ± 0.01 | 0.59 ± 0.12 | 0.22 ± 0.07 | 0.41 ± 0.11 | 0.14 ± 0.01 | 38.7 ± 1.35 |
| *French fry varieties* |  |  |  |  |  |  |  |  |  |  |  |  |  |  |  |  |  |  |  |  |  |  |
| Challenger | 2 | 2.76 ± 0.41 | 0.74 ± 0.15 | 3.43 ± 0.85 | 0.43 ± 0.18 | 0.84 ± 0.24 | 1.11 ± 0.22 | 2.15 ± 0.67 | 7.65 ± 4.07 | 0.73 ± 0.21 | 18.3 ± 5.56 | 24.2 ± 1.45 | 0.81 ± 0.29 | 13.7 ± 6.06 | 1.55 ± 0.37 | 6.51 ± 2.97 | 0.04 ± 0.01 | 1.36 ± 0.21 | 0.47 ± 0.01 | 1.14 ± 0.38 | 0.17 ± 0.05 | 88.2 ± 14.6 |
|  | 6 | 4.86 ± 3.63 | 0.69 ± 0.08 | 2.78 ± 0.12 | 0.53 ± 0.06 | 0.63 ± 0.07 | 0.82 ± 0.07 | 1.72 ± 0.23 | 7.74 ± 1.84 | 1.18 ± 0.11 | 8.77 ± 1.85 | 15.6 ± 6.76 | 0.33 ± 0.04 | 7.34 ± 2.26 | 0.88 ± 0.14 | 5.78 ± 1.43 | 0.03 ± 0.01 | 0.85 ± 0.14 | 0.70 ± 0.16 | 0.75 ± 0.03 | 0.09 ± 0.02 | 62.1 ± 6.83 |
| Daisy | 2 | 2.06 ± 0.32 | 1.04 ± 0.16 | 6.95 ± 0.84 | 1.10 ± 0.18 | 1.90 ± 0.42 | 1.31 ± 0.41 | 3.12 ± 0.71 | 7.43 ± 1.84 | 0.92 ± 0.06 | 14.1 ± 1.22 | 27.3 ± 3.15 | 1.89 ± 0.22 | 12.3 ± 0.91 | 1.88 ± 0.22 | 7.76 ± 2.63 | 0.20 ± 0.06 | 4.29 ± 2.34 | 1.24 ± 0.98 | 1.44 ± 0.54 | 0.36 ± 0.16 | 98.7 ± 11.3 |
|  | 6 | 4.25 ± 2.09 | 0.97 ± 0.41 | 6.88 ± 1.08 | 1.32 ± 0.22 | 1.63 ± 0.39 | 1.25 ± 0.19 | 3.14 ± 0.81 | 11.1 ± 1.86 | 1.70 ± 0.43 | 11.6 ± 1.94 | 17.6 ± 3.05 | 1.31 ± 0.32 | 4.43 ± 2.43 | 1.62 ± 0.26 | 6.73 ± 2.31 | 0.15 ± 0.05 | 3.45 ± 0.90 | 1.09 ± 0.10 | 1.38 ± 0.82 | 0.25 ± 0.09 | 81.8 ± 8.07 |
| Desiree | 2 | 2.24 ± 0.48 | 0.96 ± 0.21 | 7.60 ± 1.18 | 0.98 ± 0.08 | 2.51 ± 0.34 | 1.81 ± 0.38 | 3.18 ± 0.52 | 10.5 ± 4.21 | 1.18 ± 0.17 | 30.6 ± 9.14 | 25.9 ± 1.31 | 1.36 ± 0.37 | 22.8 ± 6.56 | 2.51 ± 0.33 | 6.86 ± 0.69 | 0.09 ± 0.02 | 4.06 ± 1.30 | 1.74 ± 0.42 | 4.20 ± 0.87 | 0.56 ± 0.11 | 132 ± 20.0 |
|  | 6 | 3.70 ± 1.94 | 1.06 ± 0.43 | 7.22 ± 1.87 | 1.10 ± 0.18 | 2.12 ± 0.38 | 1.76 ± 0.54 | 2.40 ± 0.67 | 9.25 ± 3.21 | 2.31 ± 0.31 | 26.4 ± 8.36 | 21.1 ± 2.91 | 0.84 ± 0.21 | 6.45 ± 1.76 | 1.71 ± 0.46 | 10.3 ± 3.87 | 0.07 ± 0.01 | 2.87 ± 0.88 | 1.40 ± 0.38 | 2.56 ± 0.50 | 0.42 ± 0.13 | 105 ± 26.3 |
| Fontane | 2 | 1.38 ± 0.00 | 0.69 ± 0.14 | 3.43 ± 0.91 | 0.47 ± 0.11 | 0.88 ± 0.25 | 0.88 ± 0.48 | 1.55 ± 0.76 | 5.82 ± 2.31 | 0.63 ± 0.14 | 10.4 ± 0.76 | 21.9 ± 3.69 | 0.54 ± 0.11 | 14.0 ± 0.49 | 0.90 ± 0.06 | 4.45 ± 2.04 | 0.11 ± 0.07 | 2.61 ± 2.51 | 0.86 ± 0.81 | 0.74 ± 0.41 | 0.33 ± 0.19 | 72.6 ± 14.5 |
|  | 6 | 2.40 ± 1.38 | 0.70 ± 0.10 | 3.43 ± 0.28 | 0.63 ± 0.14 | 0.76 ± 0.01 | 0.81 ± 0.16 | 1.73 ± 0.62 | 6.15 ± 2.06 | 1.25 ± 0.51 | 10.4 ± 1.68 | 18.8 ± 2.43 | 0.68 ± 0.03 | 12.9 ± 3.18 | 0.92 ± 0.12 | 5.64 ± 1.42 | 0.08 ± 0.01 | 1.69 ± 0.47 | 0.59 ± 0.19 | 0.74 ± 0.19 | 0.25 ± 0.02 | 70.5 ± 3.77 |
| Innovator | 2 | 1.53 ± 0.08 | 0.72 ± 0.01 | 5.16 ± 0.11 | 0.69 ± 0.05 | 1.41 ± 0.15 | 1.19 ± 0.14 | 2.71 ± 0.70 | 9.48 ± 4.23 | 1.05 ± 0.14 | 18.7 ± 3.18 | 18.4 ± 0.52 | 1.26 ± 0.34 | 13.0 ± 5.79 | 1.73 ± 0.17 | 6.71 ± 1.49 | 0.12 ± 0.03 | 2.64 ± 0.91 | 0.60 ± 0.38 | 1.55 ± 0.12 | 0.37 ± 0.04 | 89.1 ± 3.74 |
|  | 6 | 5.51 ± 2.56 | 0.92 ± 0.19 | 5.07 ± 0.51 | 0.86 ± 0.08 | 1.46 ± 0.08 | 1.28 ± 0.29 | 3.18 ± 0.19 | 7.57 ± 0.89 | 2.10 ± 0.37 | 17.8 ± 5.69 | 14.4 ± 5.97 | 0.81 ± 0.33 | 11.2 ± 2.35 | 1.27 ± 0.31 | 9.85 ± 3.65 | 0.17 ± 0.01 | 3.06 ± 1.20 | 1.06 ± 0.51 | 1.77 ± 0.08 | 0.38 ± 0.11 | 89.7 ± 10.1 |
| King Edward | 2 | 4.33 ± 1.50 | 1.46 ± 0.48 | 7.59 ± 1.00 | 1.26 ± 0.03 | 2.15 ± 0.18 | 2.13 ± 0.67 | 4.95 ± 1.07 | 9.95 ± 0.97 | 1.27 ± 0.06 | 16.4 ± 3.22 | 21.3 ± 4.02 | 1.30 ± 0.42 | 15.5 ± 3.15 | 2.52 ± 0.34 | 16.1 ± 8.66 | 0.07 ± 0.02 | 3.76 ± 1.63 | 1.83 ± 1.15 | 2.89 ± 0.33 | 0.81 ± 0.03 | 118 ± 21.3 |
|  | 6 | 6.11 ± 0.47 | 1.33 ± 0.30 | 8.59 ± 2.29 | 1.67 ± 0.30 | 2.14 ± 0.54 | 1.97 ± 0.61 | 4.88 ± 1.51 | 7.36 ± 0.72 | 3.21 ± 0.16 | 13.7 ± 6.20 | 14.7 ± 3.90 | 0.91 ± 0.28 | 8.77 ± 2.90 | 2.57 ± 0.99 | 16.6 ± 6.03 | 0.08 ± 0.04 | 2.77 ± 0.84 | 1.88 ± 0.26 | 3.00 ± 2.13 | 0.57 ± 0.21 | 103 ± 25.1 |
| Lady Blanca | 2 | 2.93 ± 0.44 | 1.17 ± 0.26 | 9.95 ± 2.20 | 0.97 ± 0.19 | 2.65 ± 0.49 | 2.21 ± 0.57 | 3.90 ± 0.99 | 11.8 ± 3.98 | 1.37 ± 0.23 | 37.5 ± 7.65 | 19.1 ± 1.90 | 1.42 ± 0.15 | 14.1 ± 3.59 | 2.81 ± 0.58 | 12.6 ± 4.74 | 0.06 ± 0.02 | 2.94 ± 0.69 | 0.98 ± 0.29 | 1.86 ± 0.63 | 0.66 ± 0.20 | 131 ± 23.1 |
|  | 6 | 6.14 ± 2.15 | 1.18 ± 0.22 | 8.15 ± 1.26 | 1.16 ± 0.13 | 1.88 ± 0.27 | 2.10 ± 0.26 | 3.67 ± 0.59 | 10.5 ± 1.00 | 2.13 ± 0.05 | 35.6 ± 4.18 | 13.3 ± 3.19 | 0.60 ± 0.13 | 9.87 ± 4.16 | 1.84 ± 0.39 | 12.1 ± 2.80 | 0.05 ± 0.01 | 2.39 ± 0.29 | 1.37 ± 0.40 | 1.23 ± 0.51 | 0.42 ± 0.05 | 116 ± 7.76 |
| Lady Olympia | 2 | 2.48 ± 0.31 | 0.90 ± 0.17 | 7.78 ± 1.66 | 1.55 ± 0.29 | 2.26 ± 0.48 | 1.12 ± 0.24 | 2.75 ± 1.39 | 6.14 ± 5.39 | 1.82 ± 0.31 | 22.3 ± 3.37 | 19.7 ± 1.84 | 1.45 ± 0.22 | 15.2 ± 3.37 | 1.84 ± 0.38 | 6.89 ± 1.76 | 0.13 ± 0.03 | 2.37 ± 0.65 | 0.77 ± 0.23 | 1.47 ± 0.56 | 0.34 ± 0.15 | 99.4 ± 14.2 |
|  | 6 | 9.77 ± 1.43 | 0.94 ± 0.24 | 9.37 ± 0.79 | 2.16 ± 0.38 | 2.66 ± 0.25 | 1.21 ± 0.16 | 3.31 ± 0.27 | 7.12 ± 0.45 | 3.47 ± 0.30 | 18.7 ± 5.62 | 8.47 ± 0.98 | 0.98 ± 0.16 | 10.4 ± 3.21 | 2.14 ± 0.04 | 6.53 ± 2.81 | 0.11 ± 0.02 | 3.23 ± 0.56 | 1.33 ± 0.85 | 1.94 ± 0.93 | 0.31 ± 0.09 | 94.3 ± 3.99 |
| Maris Piper | 2 | 1.54 ± 0.61 | 1.15 ± 0.33 | 6.31 ± 1.66 | 1.21 ± 0.15 | 2.03 ± 0.44 | 1.69 ± 0.21 | 3.58 ± 0.84 | 10.9 ± 5.08 | 1.14 ± 0.20 | 22.6 ± 5.43 | 23.3 ± 1.13 | 1.81 ± 0.74 | 15.9 ± 2.85 | 2.05 ± 0.34 | 10.2 ± 2.80 | 0.15 ± 0.04 | 4.00 ± 0.34 | 1.35 ± 0.26 | 2.01 ± 0.61 | 0.45 ± 0.09 | 113 ± 19.6 |
|  | 6 | 4.09 ± 2.25 | 0.68 ± 0.21 | 4.90 ± 1.35 | 1.05 ± 0.15 | 1.38 ± 0.28 | 1.37 ± 0.40 | 2.77 ± 1.00 | 9.76 ± 3.65 | 1.32 ± 0.26 | 17.2 ± 2.96 | 13.2 ± 4.15 | 1.22 ± 0.42 | 9.58 ± 2.49 | 1.34 ± 0.38 | 6.13 ± 1.32 | 0.10 ± 0.04 | 2.98 ± 0.86 | 0.98 ± 0.29 | 1.24 ± 0.65 | 0.27 ± 0.10 | 81.6 ± 16.5 |
| Markies | 2 | 1.48 ± 0.30 | 0.45 ± 0.07 | 2.55 ± 0.19 | 0.40 ± 0.02 | 0.65 ± 0.03 | 1.11 ± 0.31 | 1.70 ± 0.36 | 3.55 ± 1.71 | 0.63 ± 0.15 | 22.0 ± 8.10 | 21.6 ± 3.75 | 0.80 ± 0.28 | 19.7 ± 2.51 | 0.81 ± 0.05 | 6.25 ± 2.17 | 0.05 ± 0.04 | 2.68 ± 2.58 | 1.20 ± 0.40 | 0.70 ± 0.34 | 0.26 ± 0.20 | 88.6 ± 13.2 |
|  | 6 | 3.71 ± 3.92 | 0.46 ± 0.11 | 2.31 ± 0.55 | 0.41 ± 0.09 | 0.54 ± 0.20 | 0.70 ± 0.22 | 1.55 ± 0.45 | 5.88 ± 2.37 | 1.43 ± 0.26 | 16.8 ± 5.50 | 14.5 ± 6.37 | 0.66 ± 0.23 | 9.62 ± 3.36 | 0.54 ± 0.12 | 4.59 ± 1.16 | 0.03 ± 0.04 | 0.81 ± 0.24 | 0.58 ± 0.18 | 0.29 ± 0.17 | 0.09 ± 0.09 | 65.5 ± 13.3 |
| Pentland Dell | 2 | 2.61 ± 0.15 | 1.18 ± 0.16 | 6.13 ± 0.26 | 0.77 ± 0.05 | 1.67 ± 0.13 | 1.89 ± 0.29 | 4.31 ± 0.03 | 8.15 ± 3.76 | 1.45 ± 0.13 | 25.7 ± 2.19 | 31.0 ± 1.99 | 1.03 ± 0.27 | 16.3 ± 0.79 | 2.09 ± 0.25 | 8.82 ± 2.88 | 0.09 ± 0.07 | 5.07 ± 1.26 | 1.53 ± 0.48 | 2.05 ± 0.35 | 0.35 ± 0.06 | 122 ± 3.21 |
|  | 6 | 3.89 ± 1.85 | 0.71 ± 0.02 | 4.32 ± 0.41 | 0.72 ± 0.08 | 0.96 ± 0.07 | 1.14 ± 0.12 | 2.46 ± 0.33 | 7.08 ± 2.32 | 2.55 ± 0.32 | 19.4 ± 1.48 | 20.8 ± 3.20 | 0.50 ± 0.06 | 9.99 ± 3.52 | 2.30 ± 0.26 | 6.44 ± 1.23 | 0.06 ± 0.02 | 2.61 ± 0.50 | 0.72 ± 0.26 | 1.14 ± 0.38 | 0.14 ± 0.05 | 88.0 ± 8.77 |
| Ramos | 2 | 3.20 ± 0.74 | 1.47 ± 0.25 | 7.39 ± 1.55 | 0.69 ± 0.12 | 1.25 ± 0.17 | 1.78 ± 0.41 | 3.73 ± 1.33 | 9.53 ± 5.82 | 1.05 ± 0.23 | 35.3 ± 4.74 | 26.2 ± 0.33 | 2.05 ± 0.32 | 18.6 ± 5.74 | 1.53 ± 0.26 | 16.3 ± 4.60 | 0.11 ± 0.03 | 3.35 ± 0.54 | 1.26 ± 0.08 | 1.47 ± 0.40 | 0.41 ± 0.10 | 137 ± 15.5 |
|  | 6 | 6.43 ± 0.73 | 1.04 ± 0.06 | 3.82 ± 0.28 | 0.51 ± 0.02 | 0.60 ± 0.01 | 1.07 ± 0.05 | 2.21 ± 0.30 | 6.09 ± 1.41 | 1.78 ± 0.25 | 22.4 ± 5.79 | 13.6 ± 2.61 | 1.18 ± 0.21 | 8.00 ± 1.03 | 0.74 ± 0.19 | 10.8 ± 2.08 | 0.06 ± 0.05 | 2.00 ± 0.28 | 1.18 ± 0.12 | 0.44 ± 0.05 | 0.19 ± 0.01 | 84.0 ± 11.9 |
| Russet Burbank | 2 | 3.21 ± 0.59 | 1.18 ± 0.15 | 8.04 ± 1.16 | 1.04 ± 0.19 | 2.07 ± 0.39 | 1.55 ± 0.30 | 4.54 ± 0.80 | 15.6 ± 3.75 | 0.88 ± 0.13 | 37.5 ± 7.48 | 23.6 ± 1.77 | 1.62 ± 0.35 | 22.2 ± 9.03 | 2.53 ± 0.34 | 9.13 ± 2.65 | 0.09 ± 0.03 | 3.78 ± 0.58 | 1.30 ± 0.54 | 3.91 ± 1.00 | 0.69 ± 0.18 | 145 ± 24.8 |
|  | 6 | 7.69 ± 2.13 | 0.88 ± 0.15 | 6.23 ± 0.68 | 0.94 ± 0.07 | 1.44 ± 0.22 | 1.33 ± 0.26 | 3.19 ± 0.26 | 12.0 ± 1.22 | 1.59 ± 0.08 | 29.8 ± 3.00 | 13.2 ± 3.58 | 0.52 ± 0.13 | 8.12 ± 0.65 | 1.34 ± 0.10 | 7.71 ± 2.37 | 0.06 ± 0.00 | 2.34 ± 0.75 | 1.16 ± 0.39 | 1.82 ± 0.61 | 0.42 ± 0.13 | 102 ± 8.51 |
| Umatilla Russet | 2 | 2.22 ± 0.53 | 1.08 ± 0.22 | 8.36 ± 1.21 | 1.09 ± 0.15 | 2.22 ± 0.35 | 1.70 ± 0.48 | 3.68 ± 1.10 | 4.72 ± 0.79 | 1.19 ± 0.16 | 29.6 ± 4.62 | 35.6 ± 7.02 | 1.30 ± 0.06 | 16.0 ± 1.34 | 2.05 ± 0.14 | 14.9 ± 8.23 | 0.35 ± 0.19 | 5.11 ± 2.61 | 2.36 ± 1.11 | 2.14 ± 0.21 | 0.57 ± 0.13 | 136 ± 27.9 |
|  | 6 | 3.80 ± 2.03 | 0.75 ± 0.34 | 4.89 ± 2.28 | 0.89 ± 0.28 | 1.19 ± 0.33 | 0.99 ± 0.35 | 2.32 ± 0.76 | 4.46 ± 1.30 | 1.97 ± 0.44 | 19.4 ± 9.07 | 20.7 ± 8.65 | 0.56 ± 0.22 | 9.30 ± 3.82 | 1.09 ± 0.24 | 9.56 ± 6.11 | 0.13 ± 0.08 | 2.11 ± 0.90 | 0.98 ± 0.81 | 0.95 ± 0.35 | 0.28 ± 0.08 | 86.3 ± 28.7 |
| *boiling varieties* |  |  |  |  |  |  |  |  |  |  |  |  |  |  |  |  |  |  |  |  |  |  |
| Harmony | 2 | 1.32 ± 0.09 | 0.79 ± 0.06 | 5.60 ± 0.12 | 0.95 ± 0.02 | 1.71 ± 0.05 | 1.48 ± 0.13 | 2.32 ± 0.49 | 8.65 ± 3.73 | 1.21 ± 0.06 | 21.7 ± 0.88 | 24.5 ± 0.45 | 1.08 ± 0.05 | 14.7 ± 3.77 | 1.68 ± 0.07 | 5.02 ± 0.92 | 0.08 ± 0.03 | 1.56 ± 0.24 | 0.66 ± 0.11 | 0.98 ± 0.09 | 0.31 ± 0.03 | 96.4 ± 1.22 |
|  | 6 | 1.30 ± 0.30 | 0.68 ± 0.07 | 6.08 ± 0.97 | 1.48 ± 0.38 | 1.75 ± 0.41 | 1.34 ± 0.26 | 2.10 ± 0.42 | 9.13 ± 1.15 | 2.32 ± 0.43 | 13.3 ± 1.86 | 25.0 ± 2.24 | 0.62 ± 0.21 | 5.39 ± 0.85 | 1.58 ± 0.31 | 5.90 ± 1.03 | 0.05 ± 0.04 | 1.54 ± 0.40 | 1.10 ± 0.33 | 0.91 ± 0.48 | 0.30 ± 0.11 | 81.9 ± 4.52 |
